# Parental obesity and offspring cardiometabolic trajectories from childhood to adulthood

**DOI:** 10.3389/fpubh.2026.1836841

**Published:** 2026-09-28

**Authors:** Yi Qian, Chao-Nan Gao, Nai-Jun Wan, Yin-Kun Yan

**Affiliations:** 1 Department of Pediatrics, Beijing Jishuitan Hospital, Capital Medical University, Beijing, China; 2 Center for Non-communicable Disease Management, Beijing Children's Hospital, Capital Medical University, National Center for Children's Health, Beijing, China; 3 Postdoctoral Research Station for Public Health and Preventive Medicine, Hebei Medical University, Shijiazhuang, Hebei, China; 4 Department of Epidemiology and Health Statistics, School of Public Health, Hebei Medical University, Shijiazhuang, Hebei, China; 5 Hebei Key Laboratory of Environment and Human Health, Hebei Medical University, Shijiazhuang, Hebei, China

**Keywords:** cardiometabolic disorders, intergeneration association, obesity, offspring, trajectory

## Abstract

**Objectives:**

Parental obesity is a strong risk factor for childhood adiposity. However, evidence regarding its association with longitudinal cardiometabolic disease progression in offspring remains limited. We aimed to examine whether parental obesity is associated with adverse cardiometabolic trajectories in offspring from childhood to adulthood.

**Methods:**

We analyzed prospective data from the China Health and Nutrition Survey (1989–2015). Offspring aged ≥2 years were categorized according to parental overweight/obesity status: neither parent affected (Group 1, reference), only father affected (Group 2), only mother affected (Group 3), and both parents affected (Group 4). We used linear mixed-effects models with polynomial age terms to estimate longitudinal trajectories and annual changes in body mass index, waist circumference, and blood pressure. Cox proportional hazards models were used to assess hazard ratios for incident overweight/obesity, central obesity, and hypertension.

**Results:**

Among 4,982 offspring, those with both parents affected (group 4) had the highest baseline levels; however, annual rates of increase were not consistently higher. Male offspring in group 4 had the highest risks for incident overweight/obesity (HR = 3.72; 95% CI, 1.73–7.99) and central obesity (HR = 2.46; 95% CI, 1.31–4.62); the association with hypertension was elevated but did not reach statistical significance (HR = 1.55; 95% CI, 0.86–2.77). Female offspring in group 4 had the highest risk for incident overweight/obesity (HR = 4.60; 95% CI, 1.93–10.94). Formal interaction tests for sex × parental obesity were not significant for any outcome (all *P*_*interaction*_ > 0.05).

**Conclusions:**

Parental obesity, particularly when both parents are affected, is associated with higher baseline cardiometabolic risk levels and substantially higher disease risk in offspring, though the annual rates of increase do not consistently differ across groups. These findings underscore the family unit as a critical target for early preventive strategies.

## 1 Introduction

Obesity is an important public health crisis globally and in China (1, 2), and is a leading preventable cause of cardiometabolic disease, with origins often rooted in early life (3–7). Parental obesity is among the strongest known risk factors for childhood adiposity, with extensive evidence demonstrating a graded increase in risk when one or both parents are affected (8, 9). This intergenerational association is attributed to a combination of genetic predisposition (10), shared family environments (11, 12), and potential intrauterine influences (13).

While the intergenerational link regarding body mass index (BMI) is well-documented (8, 9, 14, 15), less is known about the long-term trajectories of a broader spectrum of cardiometabolic indices, such as waist circumference (WC) and blood pressure (BP), from childhood into adulthood. Furthermore, many prior studies relied on cross-sectional assessments or failed to adequately account for the dynamic nature of risk progression. Clarifying these longitudinal patterns is essential for identifying critical windows for preventive intervention. Additionally, the evidence regarding potential sex-specific differences in these trajectories remains inconsistent, and many studies lack sufficient power to distinguish between maternal and paternal contributions.

To address these gaps, we analyzed prospective data from a nationwide cohort with repeated measurements from early childhood into adulthood. Our primary aims were: (1) to characterize the longitudinal trajectories of offspring BMI, WC, and BP across distinct parental weight-status groups; (2) to determine whether parental obesity predicts an accelerated annual increase in these indices; (3) to evaluate the associated risks for incident overweight/obesity, central obesity, and hypertension, while exploring potential sexual dimorphism in these relationships.

## 2 Material and methods

### 2.1 Study design and study population

We conducted a prospective cohort analysis using data from the China Health and Nutrition Survey (CHNS), an ongoing nationwide longitudinal study initiated in 1989 (16, 17). The CHNS employs a multistage, random cluster design to recruit a representative sample across diverse geographic and socioeconomic regions in China. Data are collected in repeated waves approximately every 2–4 years and include detailed household surveys and physical examinations. The survey procedures have been described in detail elsewhere (16, 18). For the present study, we utilized data from the 1989 through 2015 survey waves. The analytical sample consisted of offspring aged ≥2 years at their baseline examination.

We included 4,982 participants who had measured anthropometric and blood pressure data at baseline, at least one follow-up assessment with the same measurements, and concurrently available measured anthropometric data for both parents. We excluded individuals with pre-existing conditions that could substantially affect growth or metabolism (e.g., type 1 diabetes, chronic kidney disease, or long-term corticosteroid use). A flowchart detailing participant selection is provided in the Supplementary Material (Supplementary Figure S1).

### 2.2 Exposure assessment

The primary exposure was parental adiposity status at the offspring's baseline, defined using two complementary criteria. First, parental overweight/obesity was classified according to BMI using Chinese criteria (19): normal weight (BMI <24 kg/m^2^), overweight (24 ≤ BMI <28 kg/m^2^), and obesity (BMI ≥28 kg/m^2^); overweight and obesity categories were combined for analysis. Second, parental central obesity was defined using WC thresholds (≥85 cm for men, ≥80 cm for women) (19). Offspring were categorized into four mutually exclusive groups: neither parent affected (Group 1, reference), only father affected (Group 2), only mother affected (Group 3), and both parents affected (Group 4).

### 2.3 Outcome assessment

Primary outcomes included repeated measures of offspring BMI, WC, systolic blood pressure (SBP), and diastolic blood pressure (DBP) across follow-up waves. All measurements were obtained by trained staff using standardized protocols (18): weight and height were measured without shoes and in lightweight clothing with calibrated instruments, WC was taken at the midpoint between the lowest rib and iliac crest. Height and WC were measured to the nearest 0.1 cm. Weight was measured to the nearest 0.1 kilogram. BMI was calculated as weight in kilograms divided by the square of height in meters. Blood pressure measurements were taken in triplicate by well-trained observers on the right arm after 10 min of seated rest using a standard mercury sphygmomanometer. Korotkoff phase 1 and phase 5 were defined as SBP and DBP, respectively. The average of the three readings was used in the analyses.

Incident disease outcomes were defined as the first occurrence of overweight/obesity, central obesity, or hypertension during follow-up. Age- and sex-specific diagnostic criteria were applied:

#### 2.3.1 Overweight/obesity

For participants aged <18 years, we used the International Obesity Task Force (IOTF) age- and sex-specific BMI cut-offs (20, 21). For participants aged ≥18 years, overweight/obesity was defined as BMI ≥24 kg/m^2^ (22);

#### 2.3.2 Central obesity

For participants aged <18 years, central obesity was defined as WC ≥90th percentile for Chinese children and adolescents (23). For adults (aged ≥18 years), central obesity was defined as WC ≥90 cm for males and ≥80 cm for females (24).

#### 2.3.3 Hypertension

For children aged <13 years, we used the 2017 AAP guidelines (based on age, sex, and height percentiles) (25). For participants aged ≥13 years, we applied the 2025 AHA/ACC thresholds (SBP ≥130 mmHg, DBP ≥80 mmHg, or reported diagnosis with antihypertensive medication use) (26).

### 2.4 Covariates

We collected data on potential confounders through structured questionnaires. Offspring-level covariates included age, sex, ethnicity (Han or other), and residential region (urban or rural). Parental-level covariates included age, educational attainment (categorized as below college or college and above), smoking status (current smoker: yes/no), and alcohol drinking status (current drinker: yes/no).

### 2.5 Statistical analysis

Data were presented as median (Q1, Q3), mean (standard deviation), or *n* (%). Sex-specific nonlinear trajectories were estimated using linear mixed-effects models containing polynomial age terms, parental-adiposity group, and their interaction, with participant-specific random effects. This approach accommodated repeated measurements and varying numbers of unequally spaced observations across individuals. Model selection was based on the Akaike Information Criterion (AIC) and the significance of polynomial age terms (*P* < 0.05) (27). Age and its polynomial terms were entered sequentially; higher-order terms were retained only if significant, did not render lower-order terms nonsignificant, and improved model fit based on AIC values. These trajectory models were descriptive and were not adjusted for sociodemographic covariates. Overall differences in age-related trajectories were assessed using joint Wald χ^2^ tests of the group × polynomial age interaction terms. For *post-hoc* analyses, all six pairwise trajectory contrasts were evaluated by jointly testing the three corresponding group × polynomial age interaction coefficients, with Holm adjustment within each outcome. Separately, average annual changes were estimated using linear mixed-effects models containing linear age, parental-adiposity group, and their interaction. All six pairwise differences in annual change were estimated with 95% CIs, with Tukey adjustment for multiple comparisons (28). Time-to-event analyses were performed using Cox proportional hazards models to estimate hazard ratios (HRs) and 95% confidence intervals (CIs) for incident outcomes. The time origin was defined as the participant's baseline examination. Participants free of the respective outcome at baseline were included in the risk set. Time-to-event was calculated from baseline to the earliest follow-up survey wave at which the incident outcome was first detected; participants without the outcome were right-censored at their last available survey wave. Covariates included offspring baseline age, ethnicity, region, and parental age, smoking status, drinking status, and education. In overall Cox models, offspring sex was additionally adjusted for; sex-stratified models did not include sex as a covariate. Formal interaction analyses were conducted by adding a parental obesity group × offspring sex interaction term to the overall Cox model; the *P* value for interaction was obtained using a likelihood ratio test comparing models with and without the interaction term.

Three distinct types of interaction analyses were conducted. First, in the nonlinear trajectory models underlying Figures 1, 2, differences in age-related trajectories across parental-adiposity groups were assessed using joint Wald tests of the group × polynomial age interaction terms. Second, average annual rates of change were estimated using linear mixed-effects models containing age as a linear continuous variable, parental-adiposity group, and their interaction, The group × linear age interaction represented the between-group difference in the average annual change over the observed age range. Third, the sex × parental obesity interaction tested whether the association between parental obesity status and incident cardiometabolic outcomes differed between males and females, assessed via the interaction term between sex and group in the Cox proportional hazards models.

**Figure 1:**
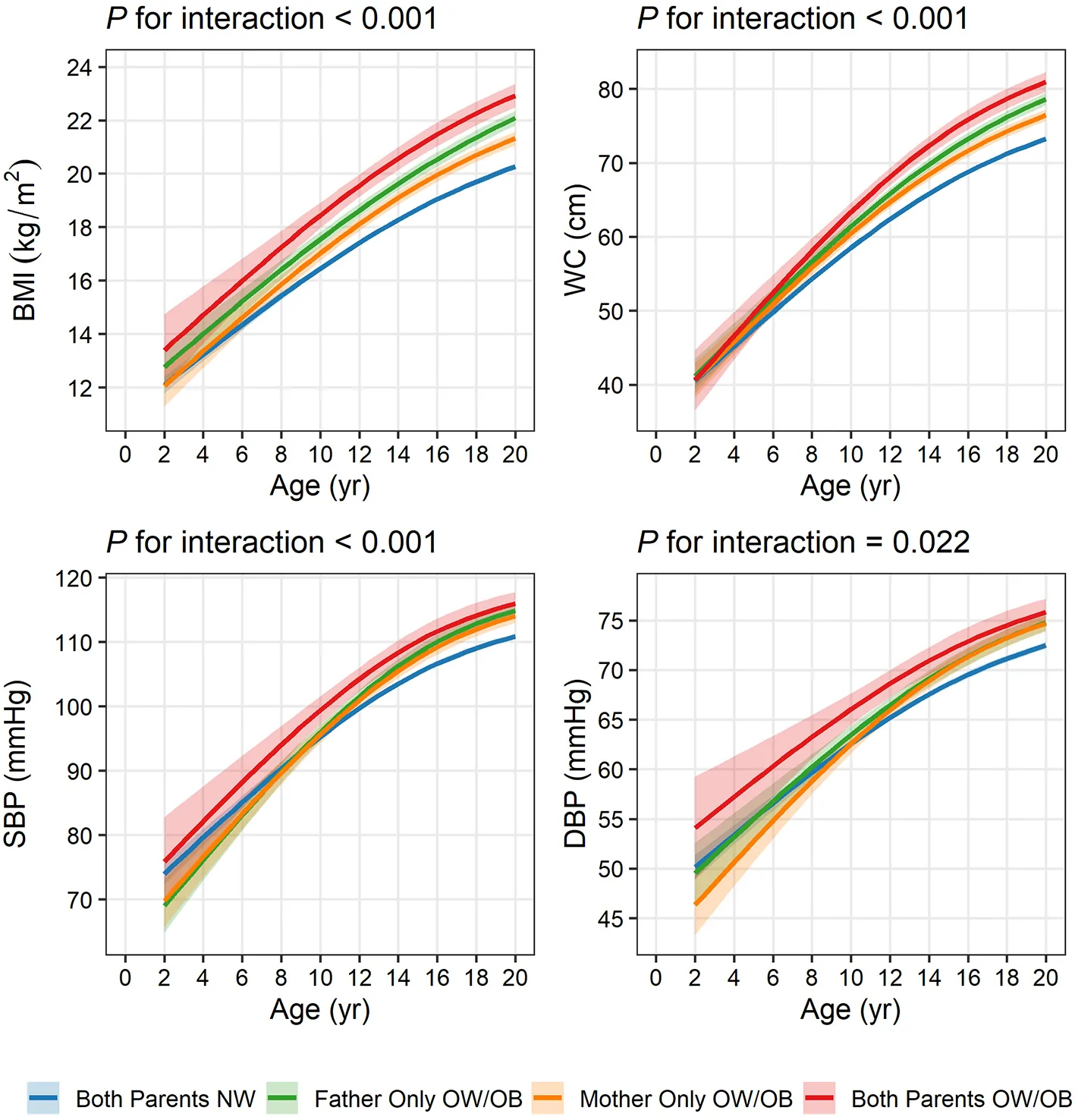
Trajectories of offspring BMI, WC, SBP, and DBP from Childhood to adulthood in boys stratified by parental weight status. BMI, body mass index; WC, waist circumference; SBP, systolic blood pressure; DBP, diastolic blood pressure; NW, normal weight; OW/OB, overweight/obese.

**Figure 2:**
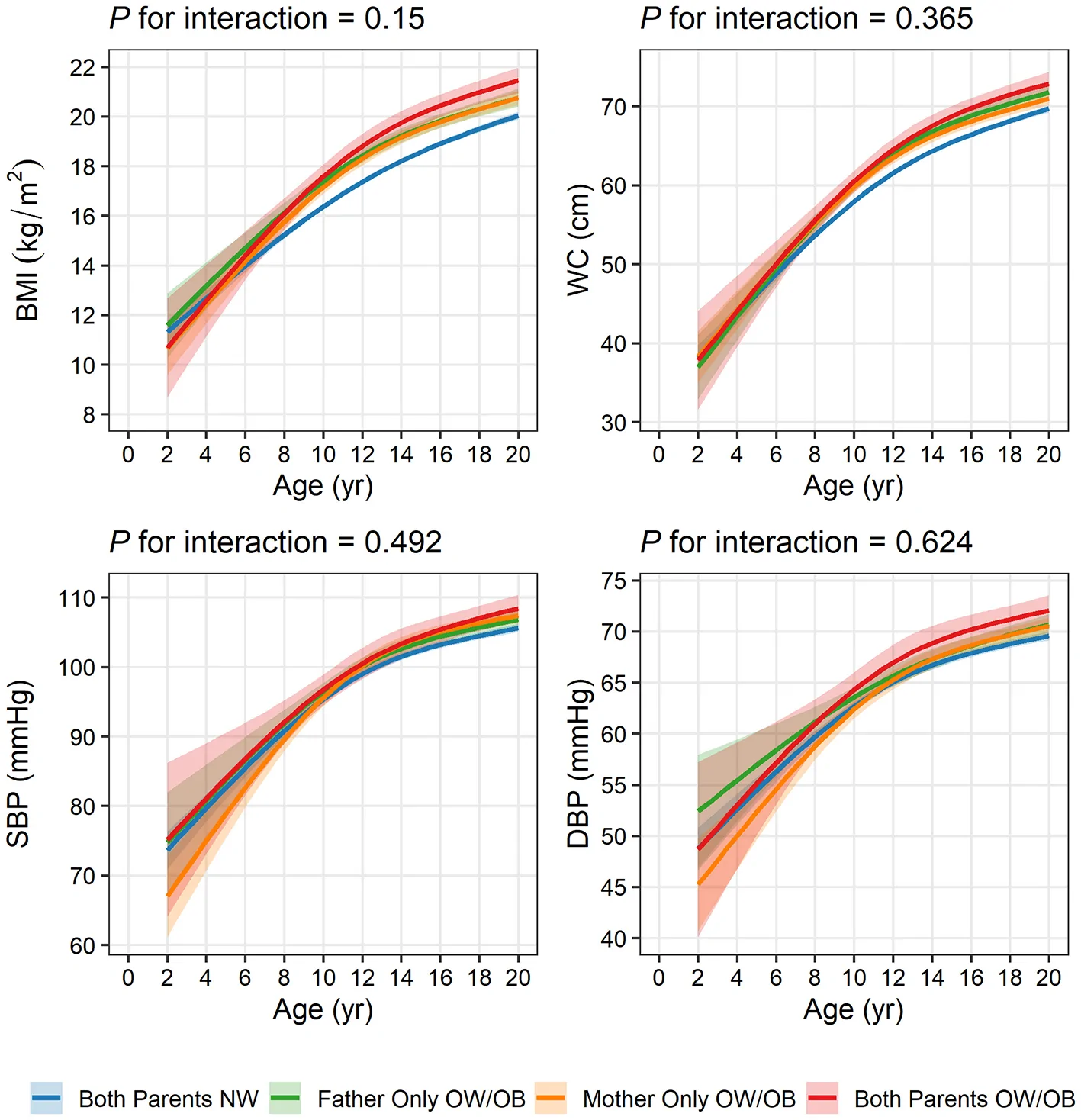
Trajectories of offspring BMI, WC, SBP, and DBP from Childhood to adulthood in girls stratified by parental weight status. BMI, body mass index; WC, waist circumference; SBP, systolic blood pressure; DBP, diastolic blood pressure. NW, normal weight; OW/OB, overweight/obese.

Additionally, for the nonlinear trajectory models, all six *post-hoc* pairwise trajectory contrasts were evaluated using joint Wald tests of the corresponding group × polynomial age interaction coefficients, with Holm adjustment within each outcome. For the linear annual-change models, all six pairwise between-group differences in annual change (Δβ) were estimated with 95% confidence intervals, with Tukey adjustment for multiple comparisons.

A two-side *P* < 0.05 was considered significant. Analyses were performed using R version 4.4.2.

## 3 Results

### 3.1 Baseline characteristics of the study population

In the overall study cohort of 4,982 participants, offspring were stratified into four groups based on parental weight status at baseline: neither parent overweight/obese (group 1, *n* = 3,346), only the father overweight/obese (group 2, *n* = 607), only the mother overweight/obese (group 3, *n* = 744), and both parents overweight/obese (group 4, *n* = 285). The comprehensive baseline characteristic are detailed in Table 1. Offspring in group 4 had the highest mean values for age (18.24 ± 9.82 years), BMI (20.71 ± 4.22 kg/m^2^), WC (70.98 ± 13.73 cm), SBP (106.19 ± 14.79 mmHg), and DBP (70.36 ± 10.43 mmHg). All inter-group comparisons for these physiological measures were statistically significant (all *P* < 0.001). The sex distribution was comparable across groups (*P* = 0.387), whereas significant differences were observed for ethnicity and residential region (both *P* < 0.001). Correspondingly, parental age, BMI, and WC were also highest in group 4 (all *P* < 0.001), with significant variations in parental education level and health-related behaviors such as smoking and alcohol consumption. Age-adjusted baseline comparisons further confirmed that Group 4 had significantly higher baseline BMI, WC, and SBP compared with Group 1 (all *P* < 0.001), with the pattern of between-group differences materially unchanged.

**Table 1:** Characteristics of the study population at baseline according to parental weight status.

Variable	G1: neither overweight/obese (*n* = 3,346)	G2: only father overweight/obese (*n* = 607)	G3: only mother overweight/obese (*n* = 744)	G4: both overweight/obese (*n* = 285)	*P*-value
Baseline offspring characteristics					
Follow-up times	3.00 (2.00, 4.00)	2.00 (2.00, 3.00)	3.00 (2.00, 3.00)	2.00 (2.00, 3.00)	<0.001
Age, years	14.50 (8.19)	14.41 (8.10)	16.09 (8.66)	18.24 (9.82)	<0.001
Sex, *n* (%)					
Males	2,051 (61.3)	365 (60.1)	431 (57.9)	175 (61.4)	0.387
Females	1,295 (38.7)	242 (39.9)	313 (42.1)	110 (38.6)	
Nationality, *n* (%)					
Han	2,801 (83.8)	553 (91.3)	664 (89.5)	263 (93.9)	
Others	542 (16.2)	53 (8.7)	78 (10.5)	17 (6.1)	<0.001
Region, *n* (%)					
Urban	950 (28.4)	234 (38.6)	273 (36.7)	126 (44.2)	<0.001
Rural	2,396 (71.6)	373 (61.4)	471 (63.3)	159 (55.8)	
BMI, kg/m^2^	17.72 (3.18)	18.93 (3.74)	18.99 (3.53)	20.71 (4.22)	<0.001
Waist circumference, cm	62.47 (10.40)	65.71 (12.48)	66.56 (11.83)	70.98 (13.73)	<0.001
SBP, mmHg	99.49 (14.16)	101.30 (15.34)	102.54 (15.44)	106.19 (14.79)	<0.001
DBP, mmHg	65.36 (10.48)	66.80 (10.36)	66.90 (10.83)	70.36 (10.43)	<0.001
Father characteristics					
Age, years	42.35 (10.44)	42.00 (9.23)	44.08 (10.69)	46.42 (10.87)	<0.001
Smoking (%)					
Yes	2,310 (69.0)	393 (64.7)	506 (68.0)	176 (61.8)	0.040
No	1,021 (30.5)	213 (35.1)	234 (31.5)	109 (38.2)	
Unknown	15 (0.4)	1 (0.2)	4 (0.5)	0 (0.0)	
Drinking (%)					
Yes	2,266 (67.7)	416 (68.5)	479 (64.4)	209 (73.3)	0.036
No	1,056 (31.6)	188 (31.0)	259 (34.8)	71 (24.9)	
Unknown	24 (0.7)	3 (0.5)	6 (0.8)	5 (1.8)	
Education, *n* (%)					
<Collage	3,256 (97.3)	567 (93.4)	711 (95.6)	269 (94.4)	<0.001
≥College	90 (2.7)	40 (6.6)	33 (4.4)	16 (5.6)	
BMI, kg/m^2^	21.34 (1.90)	26.94 (1.64)	21.79 (1.90)	27.20 (1.79)	<0.001
Waist circumference, cm	76.63 (7.15)	90.37 (7.42)	78.68 (7.69)	92.34 (7.80)	<0.001
Mother characteristics					
Age, years	40.90 (9.87)	40.45 (9.40)	42.49 (9.85)	44.70 (10.24)	<0.001
Smoking (%)					
Yes	117 (3.5)	13 (2.1)	34 (4.6)	12 (4.2)	0.331
No	3,204 (95.8)	590 (97.2)	704 (94.6)	272 (95.4)	
Unknown	25 (0.7)	4 (0.7)	6 (0.8)	1 (0.4)	
Drinking (%)					
Yes	369 (11.0)	65 (10.7)	68 (9.1)	32 (11.2)	0.728
No	2,937 (87.8)	535 (88.1)	669 (89.9)	248 (87.0)	
Unknown	40 (1.2)	7 (1.2)	7 (0.9)	5 (1.8)	
Education, *n* (%)					
<College	3,287 (98.2)	588 (96.9)	729 (98.0)	278 (97.5)	0.158
≥College	59 (1.8)	19 (3.1)	15 (2.0)	7 (2.5)	
BMI, kg/m^2^	21.36 (1.98)	21.91 (2.02)	27.15 (1.85)	27.46 (1.92)	<0.001
Waist circumference, cm	73.88 (7.03)	75.68 (7.27)	86.65 (7.81)	87.83 (7.86)	<0.001

Data were presented as median (Q1, Q3), mean (standard deviation), or *n* (%). SBP: systolic blood pressure; DBP: diastolic blood pressure.

### 3.2 Parental adiposity status and longitudinal trajectories of offspring cardiometabolic indices

The developmental trajectories of offspring BMI, WC, SBP, and DBP from childhood into adulthood, stratified by parental weight status, are illustrated in Figure 1 (males) and Figure 2 (females). In males, offspring with both parents overweight/obese (group 4) demonstrated consistently the highest trajectories for all four indices across the entire observed age span, with progressive separation from other groups over time. Those with only one affected parent (group 2, group 3) generally showed intermediate trajectories, while offspring with neither parent affected (group 1) maintained the lowest trajectories. In females, Group 4 exhibited higher baseline levels; however, the rates of change did not differ significantly from other groups, as indicated by non-significant interaction tests (*P* > 0.05 for all outcomes). *Post-hoc* comparisons of the nonlinear trajectories underlying Figure 1 showed that the significant BMI group × age interaction in males was characterized by differences between Group 4 and Group 1 (Holm-adjusted *P* = 0.001) and between Group 4 and Group 2 (Holm-adjusted *P* = 0.032). Although the overall interactions for WC and SBP were significant, no individual pairwise trajectory contrast remained significant after Holm adjustment (Supplementary Table S1). Supplementary analyses using parental central obesity yielded concordant patterns (Supplementary Figures S2 and S3). However, *post-hoc* nonlinear trajectory comparisons in males identified significant BMI differences between Group 4 and Groups 1, 2, and 3, as well as a DBP difference between Group 3 and Group 1 after Holm adjustment (Supplementary Table S4).

### 3.3 Parental adiposity status and annual rate of change in offspring outcomes

Figure 3 presents the sex-specific annual changes in BMI, WC, SBP, and DBP across parental weight-status groups. Formal pairwise contrasts for male are provided in Supplementary Table S3, Group 2 had significantly faster annual increases than Group 1 in BMI (Δβ = 0.109, 95% CI 0.038 to 0.179, *P* < 0.001), WC (Δβ = 0.291, 95% CI 0.048 to 0.534, *P* = 0.011), and SBP (Δβ = 0.481, 95% CI 0.061 to 0.901, *P* = 0.017). No other pairwise differences in annual change were significant among males, including all contrasts involving Groups 3 and 4, and no significant differences were observed for DBP. In females, none of the pairwise differences in annual change reached statistical significance for any outcome. Group-specific slope estimates with participant and observation counts are presented in Supplementary Table S2.

**Figure 3:**
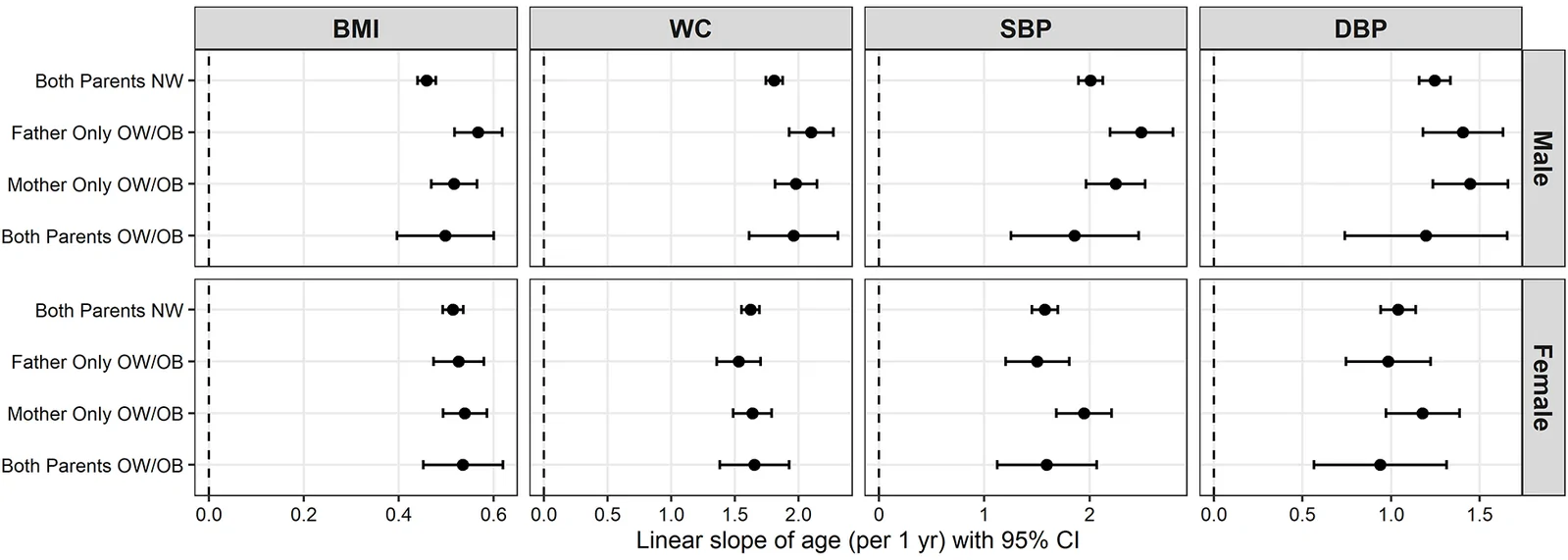
Effect of parental obesity on annual rate of change in offspring outcomes. BMI, body mass index; WC, waist circumference; SBP, systolic blood pressure; DBP, diastolic blood pressure. NW, normal weight; OW/OB, overweight/obese.

In the parallel analyses based on parental central-obesity status, no pairwise differences in average annual change were statistically significant in either males or females (Supplementary Tables S5 and S6; Supplementary Figure S4).

### 3.4 Parental adiposity status and incident disease risk in offspring

The HRs for incident overweight/obesity, central obesity, and hypertension in offspring according to parental weight status are presented in Table 2. The numbers of participants at risk, incident events, person-years, and incidence rates per 1,000 person-years for each parental weight-status group and outcome are presented in Supplementary Table S7. Compared with Group 1, male offspring in Group 4 exhibited the highest risks for incident overweight/obesity (HR = 3.72; 95% CI: 1.73–7.99) and central obesity (HR = 2.46; 95% CI: 1.31–4.62). The HR for hypertension was elevated but did not reach statistical significance (HR = 1.55; 95% CI: 0.86–2.77). Male offspring with only an affected father (Group 2) or mother (Group 3) also showed elevated risks, though generally of smaller magnitude.

**Table 2:** Hazard ratios for incident overweight/obesity, central obesity, and hypertension associated with parental weight status.

Parental obesity group	Overweight/obesity incidence	Central obesity incidence	Hypertension incidence
Overall			
G1: neither overweight/obese	1.00 (Reference)	1.00 (Reference)	1.00 (Reference)
G2: only father overweight/obese	2.34 (1.54, 3.57)	1.41 (1.04, 1.92)	1.07 (0.82, 1.41)
G3: only mother overweight/obese	1.96 (1.30, 2.96)	1.29 (0.93, 1.80)	0.95 (0.72, 1.24)
G4: both parents overweight/obese	3.87 (2.22, 6.77)	1.83 (1.19, 2.83)	1.47 (0.97, 2.21)
Boys			
G1: neither overweight/obese	1.00 (Reference)	1.00 (Reference)	1.00 (Reference)
G2: only father overweight/obese	1.91 (1.10, 3.32)	1.61 (1.02, 2.53)	1.17 (0.82, 1.68)
G3: only mother overweight/obese	2.13 (1.26, 3.59)	1.10 (0.64, 1.88)	1.13 (0.80, 1.60)
G4: both parents overweight/obese	3.72 (1.73, 7.99)	2.46 (1.31, 4.62)	1.55 (0.86, 2.77)
Girls			
G1: neither overweight/obese	1.00 (Reference)	1.00 (Reference)	1.00 (Reference)
G2: only father overweight/obese	3.65 (1.83, 7.29)	1.37 (0.89, 2.12)	0.98 (0.64, 1.51)
G3: only mother overweight/obese	1.67 (0.84, 3.31)	1.50 (0.98, 2.27)	0.79 (0.51, 1.20)
G4: both parents overweight/obese	4.60 (1.93, 10.94)	1.45 (0.77, 2.73)	1.33 (0.74, 2.41)
$P_{\text{interaction }\left(\text{se}{\text{x}}^{*}parental\;obesity\right)}$	0.757	0.356	0.541

Covariates included sex (for overall model), offspring baseline age, ethnicity, region, parental age, smoking status, drinking status, and education at the survey year. Participants at risk, incident event counts, person-years, and incidence rates according to parental weight-status group and outcome are provided in Supplementary Table S7. ^*^P values for the interaction between offspring sex and parental weight status (sex × parental obesity interaction), tested by adding a cross-product term to the Cox proportional hazards models. *P* < 0.05 indicates a significant sex difference in the association between parental obesity and offspring outcomes.

Among female offspring, Group 4 carried the highest risk for incident overweight/obesity (HR = 4.60; 95% CI: 1.93–10.94). However, associations with incident central obesity (Group 2: HR=1.37, 95% CI: 0.89-2.12; Group 4: HR=1.45, 95% CI: 0.77–2.73) were not statistically significant, and no significant associations were observed for hypertension in any female subgroup (all 95% CIs crossing 1.0).

Formal interaction tests between offspring sex and parental obesity group were not significant for any outcome (obesity: *P* = 0.757; central obesity: *P* = 0.356; hypertension: *P* = 0.541), indicating that the observed sex-stratified differences should be interpreted as exploratory rather than definitive. Supplementary analysis using parental central obesity showed a largely consistent pattern, with particularly strong risks for obesity when both parents had central obesity (Supplementary Table S8). The corresponding numbers of participants at risk, incident events, person-years, and incidence rates are presented in Supplementary Table S9.

## 4 Discussion

This large, prospective cohort study, utilizing repeated measurements spanning from early childhood into adulthood, provides further evidence supporting an association between parental overweight/obesity and adverse cardiometabolic trajectories in offspring, characterized primarily by elevated baseline levels rather than consistently accelerated annual progression. These findings align with and extend previous literature (14, 29) by illustrating the longitudinal course of risk accrual in a Chinese population and recognizing the family unit, particularly the combined weight status of both parents, as a critical determinant and a potent early-life risk identifier.

The observed graded increase in risk from no affected parent to one, and to both suggests a potential dose-response cumulative burden stemming from the interplay of genetic predisposition and a shared familial environment that promotes obesogenic behaviors. By mapping the dynamic progression of risk over time, we demonstrate that parental obesity predicts not just a static state of adiposity but a process of risk accrual that is characterized primarily by elevated baseline levels rather than consistently accelerated annual progression. This underscores the importance of interventions aimed not only at reducing the absolute level of a risk factor but, crucially, at flattening the trajectory of its progression from an early age.

The graded increase in offspring cardiometabolic risk with parental adiposity burden observed in this study suggests that genetic susceptibility and early-life biological programming may play important roles in the development of childhood obesity. Children of two affected parents inherit a doubled genetic load, substantially increasing polygenic risk for obesity and metabolic dysregulation. Mendelian randomization studies suggest that parental adiposity may influence offspring through genetic transmission, while maternal obesity may additionally act through intrauterine pathways (30–32). In our study, however, the most consistent finding was the markedly higher risk of incident overweight/obesity among offspring with both parents affected, in both the overall and sex-stratified analyses. This pattern may reflect the cumulative influence of genetic susceptibility and shared obesogenic family environments. Although father-only estimates were numerically higher than mother-only estimates across all three outcomes in the overall models, this pattern was not consistent in the sex-stratified analyses, and the confidence intervals overlapped substantially. Therefore, our findings do not support conclusions regarding parent-specific mechanisms. Regarding average annual progression, significant differences were limited to greater increases in BMI, WC, and SBP in males in Group 2 compared with Group 1; Group 4 did not differ significantly from Group 1. Further studies with larger samples are needed to disentangle maternal and paternal contributions, and these sex-stratified observations should be considered exploratory, and any sex-specific biological interpretations remain speculative at this stage.

Notably, in households with two obese parents, obesogenic risk factors, including encompassing energy-dense diets, sedentary routines, and physical inactivity, were more concentrated, and children may consistently model these lifestyle behaviors. Chronic exposure to such environmental influences may interact with genetic susceptibility, potentially underpinning the higher baseline levels of cardiometabolic indices observed in offspring of parents with obesity. Nevertheless, we acknowledge that we did not have detailed longitudinal data on diet, physical activity, or pubertal development, and residual confounding from these unmeasured family-level factors may influence our findings.

Formal between-group contrasts showed limited and outcome-specific differences in average annual progression. In males, Group 2 showed significantly greater annual increases than Group 1 in BMI, WC, and SBP, whereas no other pairwise differences were significant. Because Group 2 did not differ from Groups 3 or 4, this finding should not be interpreted as evidence of a stronger paternal effect. In contrast, the higher incident disease risks in Group 4 were not accompanied by significantly faster average annual progression, suggesting that cardiometabolic differences established earlier in life may be more important than consistently accelerated linear change. However, the wide confidence intervals in Group 4 warrant cautious interpretation.

Previous systematic review and meta-analysis studies have extensively examined the role of maternal weight status both before and during pregnancy in shaping offspring BP (33, 34). Our finding that hypertension risk was elevated but not statistically significant in either sex, despite visible trajectory differences, suggests that blood pressure may be more strongly influenced by concurrent adult weight status or other time-varying factors than by early-life parental adiposity alone. Alternatively, the relatively modest number of hypertension events in the dual-exposed group may have limited statistical power to detect a significant hazard.

Our study has several strengths, including its prospective design, large, nationally representative sample, long follow-up from school age into early adulthood, use of objectively measured anthropometric and clinical data, and examination of both continuous trajectories and incident disease. These findings provided meaningful clinical and public health relevance. Assessment of parental weight status may offer? a pragmatic, low-cost approach for initial stratification of offspring cardiometabolic risk. Offspring of two obese parents may constitute a distinct high-risk subgroup that warrants consideration for prioritized intensified monitoring and early family-centered lifestyle interventions. Our trajectory analyses suggest that childhood may represent a plausible window for effective intervention, with the potential to prevent further worsening of an already elevated cardiometabolic risk profile. Preliminary evidence further indicates that adult obesity-prevention programs may yield intergenerational benefits, improving parental health while potentially modifying offspring lifelong cardiometabolic trajectories.

Several limitations should be considered. First, the analytical sample included participants with a broad baseline age range (≥2 years), and the baseline age distribution differed across parental groups, particularly with the highest mean age in the dual-affected group. Although statistical adjustments were made, age-related biases may influence the estimated trajectories. Although Group 4 participants were on average nearly 4 years older at baseline than Group 1, the between-group differences in baseline cardiometabolic measures persisted after adjustment for baseline age, and the prospective Cox models also included baseline age as a continuous covariate. Nevertheless, residual confounding related to age at cohort entry cannot be completely excluded. Second, the characterization of parental adiposity was based on baseline measurements only; changes over time were not captured, which could lead to exposure misclassification. Third, despite adjustment for key sociodemographic confounders, residual confounding by unmeasured or imprecisely measured factors (e.g., detailed dietary intake, physical activity, pubertal development, and genetic data) is possible. Fourth, our sex-stratified findings showed overlapping confidence intervals and non-significant formal interaction tests (all *P* > 0.05), which precludes strong conclusions about sexual dimorphism. We have therefore refrained from claiming definitive sex differences and present these findings as exploratory. Finally, the findings are based on a Chinese population, and their generalizability to other ethnic and cultural contexts requires confirmation. Future studies are warranted to conduct family-based randomized controlled trials and population-based large-scale cohort studies with refined confounder measurements to validate these findings and substantiate the potential causal associations.

In conclusion, this longitudinal study demonstrates that parental obesity, especially when both parents are affected, identifies children who are on an adverse cardiometabolic risk profile established early, with baseline differences being the predominant driver from childhood onward. These findings reinforce the utility of assessing parental weight status as a simple tool for early risk stratification, while also noting that exploratory sex-stratified analyses generated hypotheses that require confirmation in larger cohorts with formal interaction testing.

## Data Availability

The original contributions presented in the study are included in the article/Supplementary material, further inquiries can be directed to the corresponding authors.
