# Supplementary material for: Parental obesity and offspring cardiometabolic trajectories from childhood to adulthood
Source: Front Public Health. 2026 Sep 28;14:1836841. doi: 10.3389/fpubh.2026.1836841 (PMC13654761; doi:10.3389/fpubh.2026.1836841)
Supplement: Supplementary file 1 [file Table_1.DOCX]

**Figure S1. Flow chart of selection of study participants.**

**Figure S2. Trajectories of offspring BMI, WC, SBP, and DBP from childhood to adulthood in boys, stratified by parental central obesity.**

BMI: body mass index; WC: waist circumference; SBP:systolic blood pressure; DBP: diastolic blood pressure.

**Figure S3. Trajectories of offspring BMI, WC, SBP, and DBP from childhood to adulthood in girls, stratified by parental central obesity.**

BMI: body mass index; WC: waist circumference; SBP:systolic blood pressure; DBP: diastolic blood pressure.

**Figure S4. Effect of parental central obesity on annual rate of change in offspring outcomes.**

BMI: body mass index; WC: waist circumference; SBP:systolic blood pressure; DBP: diastolic blood pressure.

**Supplementary Table S1. Post-hoc pairwise comparisons of age-related trajectories among parental weight-status groups in males**

| **Outcome** | **Overall interaction *P*** | **Group contrast** | **Wald χ²** | **df** | **Unadjusted *P*** | **Holm-adjusted *P*** |
| --- | --- | --- | --- | --- | --- | --- |
| **BMI** | <0.001 | Group 2 vs Group 1 | 6.218 | 3 | 0.101 | 0.203 |
|  |  | Group 3 vs Group 1 | 7.416 | 3 | **0.060** | 0.179 |
|  |  | Group 4 vs Group 1 | 19.769 | 3 | **<0.001** | **0.001** |
|  |  | Group 3 vs Group 2 | 2.707 | 3 | 0.439 | 0.439 |
|  |  | Group 4 vs Group 2 | 12.334 | 3 | **0.006** | **0.032** |
|  |  | Group 4 vs Group 3 | 8.686 | 3 | **0.034** | 0.135 |
| **WC** | 0.001 | Group 2 vs Group 1 | 7.919 | 3 | **0.048** | 0.286 |
|  |  | Group 3 vs Group 1 | 7.027 | 3 | **0.071** | 0.321 |
|  |  | Group 4 vs Group 1 | 7.255 | 3 | **0.064** | 0.321 |
|  |  | Group 3 vs Group 2 | 5.770 | 3 | 0.123 | 0.370 |
|  |  | Group 4 vs Group 2 | 2.442 | 3 | 0.486 | 0.972 |
|  |  | Group 4 vs Group 3 | 1.810 | 3 | 0.613 | 0.972 |
| **SBP** | <0.001 | Group 2 vs Group 1 | 6.687 | 3 | **0.083** | 0.495 |
|  |  | Group 3 vs Group 1 | 4.810 | 3 | 0.186 | 0.841 |
|  |  | Group 4 vs Group 1 | 1.994 | 3 | 0.574 | 1.000 |
|  |  | Group 3 vs Group 2 | 0.326 | 3 | 0.955 | 1.000 |
|  |  | Group 4 vs Group 2 | 5.050 | 3 | 0.168 | 0.841 |
|  |  | Group 4 vs Group 3 | 3.763 | 3 | 0.288 | 0.865 |
| **DBP** | 0.022 | Group 2 vs Group 1 | 2.760 | 3 | 0.430 | 1.000 |
|  |  | Group 3 vs Group 1 | 10.222 | 3 | **0.017** | 0.101 |
|  |  | Group 4 vs Group 1 | 0.527 | 3 | 0.913 | 1.000 |
|  |  | Group 3 vs Group 2 | 3.166 | 3 | 0.367 | 1.000 |
|  |  | Group 4 vs Group 2 | 1.505 | 3 | 0.681 | 1.000 |
|  |  | Group 4 vs Group 3 | 3.706 | 3 | 0.295 | 1.000 |

**Notes:** Group 1, both parents with normal weight; Group 2, father only with overweight/obesity; Group 3, mother only with overweight/obesity; Group 4, both parents with overweight/obesity. P values were adjusted for six pairwise comparisons within each outcome using the Holm method. Bold values indicate Holm-adjusted P < 0.05. Participant/observation counts were: Group 1, 1,163/2,979; Group 2, 211/530; Group 3, 198/494; and Group 4, 69/163.

**Interpretation:** For BMI, the Group 4 versus Group 1 and Group 4 versus Group 2 trajectory contrasts remained significant after Holm adjustment. No individual WC, SBP, or DBP pairwise trajectory contrast remained significant after adjustment.

**Supplementary Table S2. Sex-specific annual changes in cardiometabolic outcomes by parental weight-status group, with participant and observation counts**

| **Outcome** | **Group** | **Parental weight-status category** | **Participants, n** | **Observations, n** | **Annual change, β (95% CI)** |
| --- | --- | --- | --- | --- | --- |
| ***Male*** | | | | | |
| BMI | Group 1 | Both Parents NW | 1,095 | 2,794 | 0.459 (0.440, 0.478) |
| BMI | Group 2 | Father Only OW/OB | 176 | 438 | 0.568 (0.517, 0.618) |
| BMI | Group 3 | Mother Only OW/OB | 178 | 442 | 0.517 (0.469, 0.565) |
| BMI | Group 4 | Both Parents OW/OB | 50 | 119 | 0.498 (0.396, 0.600) |
| WC | Group 1 | Both Parents NW | 1,095 | 2,794 | 1.808 (1.741, 1.874) |
| WC | Group 2 | Father Only OW/OB | 176 | 438 | 2.099 (1.926, 2.272) |
| WC | Group 3 | Mother Only OW/OB | 178 | 442 | 1.980 (1.815, 2.145) |
| WC | Group 4 | Both Parents OW/OB | 50 | 119 | 1.959 (1.611, 2.308) |
| SBP | Group 1 | Both Parents NW | 1,095 | 2,794 | 2.010 (1.895, 2.126) |
| SBP | Group 2 | Father Only OW/OB | 176 | 438 | 2.492 (2.193, 2.791) |
| SBP | Group 3 | Mother Only OW/OB | 178 | 442 | 2.247 (1.965, 2.528) |
| SBP | Group 4 | Both Parents OW/OB | 50 | 119 | 1.857 (1.251, 2.464) |
| DBP | Group 1 | Both Parents NW | 1,095 | 2,794 | 1.246 (1.159, 1.334) |
| DBP | Group 2 | Father Only OW/OB | 176 | 438 | 1.406 (1.180, 1.632) |
| DBP | Group 3 | Mother Only OW/OB | 178 | 442 | 1.447 (1.235, 1.659) |
| DBP | Group 4 | Both Parents OW/OB | 50 | 119 | 1.197 (0.739, 1.655) |
| ***Female*** | | | | | |
| BMI | Group 1 | Both Parents NW | 950 | 2,365 | 0.514 (0.493, 0.536) |
| BMI | Group 2 | Father Only OW/OB | 165 | 403 | 0.526 (0.473, 0.579) |
| BMI | Group 3 | Mother Only OW/OB | 195 | 490 | 0.539 (0.493, 0.586) |
| BMI | Group 4 | Both Parents OW/OB | 61 | 149 | 0.535 (0.451, 0.619) |
| WC | Group 1 | Both Parents NW | 950 | 2,365 | 1.622 (1.551, 1.692) |
| WC | Group 2 | Father Only OW/OB | 165 | 403 | 1.528 (1.355, 1.701) |
| WC | Group 3 | Mother Only OW/OB | 195 | 490 | 1.637 (1.486, 1.788) |
| WC | Group 4 | Both Parents OW/OB | 61 | 149 | 1.652 (1.380, 1.925) |
| SBP | Group 1 | Both Parents NW | 950 | 2,365 | 1.574 (1.450, 1.699) |
| SBP | Group 2 | Father Only OW/OB | 165 | 403 | 1.503 (1.201, 1.805) |
| SBP | Group 3 | Mother Only OW/OB | 195 | 490 | 1.945 (1.683, 2.208) |
| SBP | Group 4 | Both Parents OW/OB | 61 | 149 | 1.594 (1.122, 2.065) |
| DBP | Group 1 | Both Parents NW | 950 | 2,365 | 1.039 (0.940, 1.138) |
| DBP | Group 2 | Father Only OW/OB | 165 | 403 | 0.983 (0.744, 1.223) |
| DBP | Group 3 | Mother Only OW/OB | 195 | 490 | 1.178 (0.970, 1.387) |
| DBP | Group 4 | Both Parents OW/OB | 61 | 149 | 0.939 (0.564, 1.313) |

*Note.* β represents the model-estimated annual change in the original unit of each outcome. Participant and observation counts are based on records actually included in the corresponding mixed-effects model. BMI = body mass index; WC = waist circumference; SBP = systolic blood pressure; DBP = diastolic blood pressure; CI = confidence interval; NW = normal weight; OW/OB = overweight/obese. Group definitions and abbreviations are provided in Supplementary Table S1.

**Supplementary Table S3. Pairwise between-group differences in annual changes in cardiometabolic outcomes**

| **Outcome** | **Contrast** | **Difference in annual change, Δβ (95% CI)** | **Adjusted *P* value** |
| --- | --- | --- | --- |
| ***Male*** | | | |
| BMI | Group 2 - Group 1 | 0.109 (0.038, 0.179) | <0.001 |
| BMI | Group 3 - Group 1 | 0.058 (-0.010, 0.126) | 0.127 |
| BMI | Group 4 - Group 1 | 0.039 (-0.097, 0.175) | 0.881 |
| BMI | Group 3 - Group 2 | -0.051 (-0.142, 0.040) | 0.481 |
| BMI | Group 4 - Group 2 | -0.070 (-0.218, 0.079) | 0.626 |
| BMI | Group 4 - Group 3 | -0.019 (-0.166, 0.129) | 0.988 |
| WC | Group 2 - Group 1 | 0.291 (0.048, 0.534) | 0.011 |
| WC | Group 3 - Group 1 | 0.172 (-0.060, 0.405) | 0.227 |
| WC | Group 4 - Group 1 | 0.152 (-0.313, 0.617) | 0.836 |
| WC | Group 3 - Group 2 | -0.119 (-0.432, 0.194) | 0.764 |
| WC | Group 4 - Group 2 | -0.139 (-0.649, 0.371) | 0.897 |
| WC | Group 4 - Group 3 | -0.020 (-0.526, 0.485) | 1.000 |
| SBP | Group 2 - Group 1 | 0.481 (0.061, 0.901) | 0.017 |
| SBP | Group 3 - Group 1 | 0.236 (-0.163, 0.635) | 0.425 |
| SBP | Group 4 - Group 1 | -0.153 (-0.962, 0.656) | 0.962 |
| SBP | Group 3 - Group 2 | -0.245 (-0.783, 0.293) | 0.646 |
| SBP | Group 4 - Group 2 | -0.634 (-1.521, 0.252) | 0.255 |
| SBP | Group 4 - Group 3 | -0.389 (-1.266, 0.487) | 0.664 |
| DBP | Group 2 - Group 1 | 0.160 (-0.158, 0.477) | 0.568 |
| DBP | Group 3 - Group 1 | 0.201 (-0.100, 0.502) | 0.316 |
| DBP | Group 4 - Group 1 | -0.049 (-0.661, 0.562) | 0.997 |
| DBP | Group 3 - Group 2 | 0.041 (-0.365, 0.447) | 0.994 |
| DBP | Group 4 - Group 2 | -0.209 (-0.879, 0.460) | 0.853 |
| DBP | Group 4 - Group 3 | -0.250 (-0.912, 0.411) | 0.766 |
| ***Female*** | | | |
| BMI | Group 2 - Group 1 | 0.012 (-0.063, 0.087) | 0.977 |
| BMI | Group 3 - Group 1 | 0.025 (-0.042, 0.092) | 0.773 |
| BMI | Group 4 - Group 1 | 0.021 (-0.093, 0.135) | 0.964 |
| BMI | Group 3 - Group 2 | 0.013 (-0.079, 0.106) | 0.983 |
| BMI | Group 4 - Group 2 | 0.009 (-0.121, 0.140) | 0.998 |
| BMI | Group 4 - Group 3 | -0.004 (-0.130, 0.122) | 1.000 |
| WC | Group 2 - Group 1 | -0.094 (-0.338, 0.151) | 0.760 |
| WC | Group 3 - Group 1 | 0.015 (-0.203, 0.234) | 0.998 |
| WC | Group 4 - Group 1 | 0.031 (-0.339, 0.400) | 0.997 |
| WC | Group 3 - Group 2 | 0.109 (-0.192, 0.410) | 0.789 |
| WC | Group 4 - Group 2 | 0.124 (-0.299, 0.547) | 0.875 |
| WC | Group 4 - Group 3 | 0.015 (-0.393, 0.424) | 1.000 |
| SBP | Group 2 - Group 1 | -0.071 (-0.499, 0.357) | 0.974 |
| SBP | Group 3 - Group 1 | 0.371 (-0.010, 0.752) | 0.059 |
| SBP | Group 4 - Group 1 | 0.019 (-0.620, 0.659) | 1.000 |
| SBP | Group 3 - Group 2 | 0.442 (-0.082, 0.967) | 0.133 |
| SBP | Group 4 - Group 2 | 0.090 (-0.644, 0.824) | 0.989 |
| SBP | Group 4 - Group 3 | -0.352 (-1.060, 0.356) | 0.577 |
| DBP | Group 2 - Group 1 | -0.056 (-0.395, 0.284) | 0.975 |
| DBP | Group 3 - Group 1 | 0.140 (-0.163, 0.442) | 0.637 |
| DBP | Group 4 - Group 1 | -0.100 (-0.608, 0.407) | 0.957 |
| DBP | Group 3 - Group 2 | 0.195 (-0.221, 0.612) | 0.624 |
| DBP | Group 4 - Group 2 | -0.044 (-0.627, 0.538) | 0.997 |
| DBP | Group 4 - Group 3 | -0.240 (-0.801, 0.322) | 0.692 |

*Note.* Δβ is the annual-change estimate for the first group minus that for the second group; positive values indicate a steeper annual increase in the first group. All six pairwise contrasts are shown for each sex and outcome. Confidence intervals and P values were adjusted for multiple pairwise comparisons using the Tukey method. Group definitions and abbreviations are provided in Supplementary Table S1.

**Supplementary Table S4. Post-hoc pairwise comparisons of age-related trajectories among parental central-obesity groups in males**

| **Outcome** | **Overall interaction P** | **Group contrast** | **Wald χ²** | **df** | **Unadjusted P** | **Holm-adjusted P** |
| --- | --- | --- | --- | --- | --- | --- |
| **BMI** | 0.003 | Group 2 vs Group 1 | 4.766 | 3 | 0.190 | 0.380 |
|  |  | Group 3 vs Group 1 | 8.097 | 3 | 0.044 | 0.132 |
|  |  | Group 4 vs Group 1 | 35.761 | 3 | <0.001 | **<0.001** |
|  |  | Group 3 vs Group 2 | 2.021 | 3 | 0.568 | 0.568 |
|  |  | Group 4 vs Group 2 | 16.161 | 3 | 0.001 | **0.004** |
|  |  | Group 4 vs Group 3 | 18.180 | 3 | <0.001 | **0.002** |
| **WC** | <0.001 | Group 2 vs Group 1 | 3.348 | 3 | 0.341 | 0.961 |
|  |  | Group 3 vs Group 1 | 5.864 | 3 | 0.118 | 0.592 |
|  |  | Group 4 vs Group 1 | 5.302 | 3 | 0.151 | 0.604 |
|  |  | Group 3 vs Group 2 | 2.871 | 3 | 0.412 | 0.961 |
|  |  | Group 4 vs Group 2 | 3.503 | 3 | 0.320 | 0.961 |
|  |  | Group 4 vs Group 3 | 6.807 | 3 | 0.078 | 0.470 |
| **SBP** | 0.468 | Group 2 vs Group 1 | 3.439 | 3 | 0.329 | 1.000 |
|  |  | Group 3 vs Group 1 | 9.682 | 3 | 0.021 | 0.129 |
|  |  | Group 4 vs Group 1 | 2.662 | 3 | 0.447 | 1.000 |
|  |  | Group 3 vs Group 2 | 0.584 | 3 | 0.900 | 1.000 |
|  |  | Group 4 vs Group 2 | 2.875 | 3 | 0.411 | 1.000 |
|  |  | Group 4 vs Group 3 | 2.515 | 3 | 0.473 | 1.000 |
| **DBP** | 0.724 | Group 2 vs Group 1 | 4.386 | 3 | 0.223 | 1.000 |
|  |  | Group 3 vs Group 1 | 15.508 | 3 | 0.001 | **0.009** |
|  |  | Group 4 vs Group 1 | 2.062 | 3 | 0.560 | 1.000 |
|  |  | Group 3 vs Group 2 | 1.491 | 3 | 0.684 | 1.000 |
|  |  | Group 4 vs Group 2 | 1.190 | 3 | 0.755 | 1.000 |
|  |  | Group 4 vs Group 3 | 0.595 | 3 | 0.898 | 1.000 |

**Notes:** Group 1, neither parent with central obesity; Group 2, father only with central obesity; Group 3, mother only with central obesity; Group 4, both parents with central obesity. P values were adjusted for six pairwise comparisons within each outcome using the Holm method. Bold values indicate Holm-adjusted P < 0.05. Participant/observation counts were: Group 1, 1,060/2,720; Group 2, 129/328; Group 3, 359/907; and Group 4, 93/211. Overall interaction P values were not included in the supplied pairwise-comparison file and are therefore shown as em dashes.

**Interpretation:** For BMI, the Group 4 versus Group 1, Group 4 versus Group 2, and Group 4 versus Group 3 trajectory contrasts remained significant after Holm adjustment. For DBP, the Group 3 versus Group 1 trajectory contrast remained significant after adjustment. No individual WC or SBP pairwise trajectory contrast remained significant after adjustment.

**Supplementary Table S5. Sex-specific annual changes in cardiometabolic outcomes by parental central-obesity group, with participant and observation counts**

| **Outcome** | **Group** | **Parental central-obesity category** | **Participants, n** | **Observations, n** | **Annual change, β (95% CI)** |
| --- | --- | --- | --- | --- | --- |
| ***Male*** | | | | | |
| BMI | Group 1 | Both Parents Non-central | 1,042 | 2,673 | 0.443 (0.422, 0.465) |
| BMI | Group 2 | Father Only Central Obesity | 121 | 310 | 0.522 (0.459, 0.584) |
| BMI | Group 3 | Mother Only Central Obesity | 340 | 864 | 0.454 (0.416, 0.492) |
| BMI | Group 4 | Both Parents Central Obesity | 80 | 184 | 0.453 (0.359, 0.546) |
| WC | Group 1 | Both Parents Non-central | 1,042 | 2,673 | 1.858 (1.789, 1.928) |
| WC | Group 2 | Father Only Central Obesity | 121 | 310 | 2.115 (1.914, 2.316) |
| WC | Group 3 | Mother Only Central Obesity | 340 | 864 | 1.944 (1.821, 2.067) |
| WC | Group 4 | Both Parents Central Obesity | 80 | 184 | 2.113 (1.819, 2.407) |
| SBP | Group 1 | Both Parents Non-central | 1,042 | 2,673 | 2.030 (1.911, 2.149) |
| SBP | Group 2 | Father Only Central Obesity | 121 | 310 | 2.160 (1.817, 2.504) |
| SBP | Group 3 | Mother Only Central Obesity | 340 | 864 | 2.129 (1.921, 2.337) |
| SBP | Group 4 | Both Parents Central Obesity | 80 | 184 | 1.842 (1.351, 2.333) |
| DBP | Group 1 | Both Parents Non-central | 1,042 | 2,673 | 1.288 (1.199, 1.378) |
| DBP | Group 2 | Father Only Central Obesity | 121 | 310 | 1.156 (0.897, 1.415) |
| DBP | Group 3 | Mother Only Central Obesity | 340 | 864 | 1.259 (1.102, 1.415) |
| DBP | Group 4 | Both Parents Central Obesity | 80 | 184 | 1.436 (1.067, 1.806) |
| ***Female*** | | | | | |
| BMI | Group 1 | Both Parents Non-central | 866 | 2,173 | 0.492 (0.467, 0.517) |
| BMI | Group 2 | Father Only Central Obesity | 103 | 266 | 0.455 (0.381, 0.529) |
| BMI | Group 3 | Mother Only Central Obesity | 339 | 835 | 0.512 (0.473, 0.552) |
| BMI | Group 4 | Both Parents Central Obesity | 81 | 195 | 0.461 (0.379, 0.543) |
| WC | Group 1 | Both Parents Non-central | 866 | 2,173 | 1.661 (1.589, 1.734) |
| WC | Group 2 | Father Only Central Obesity | 103 | 266 | 1.706 (1.489, 1.923) |
| WC | Group 3 | Mother Only Central Obesity | 339 | 835 | 1.714 (1.599, 1.830) |
| WC | Group 4 | Both Parents Central Obesity | 81 | 195 | 1.804 (1.568, 2.041) |
| SBP | Group 1 | Both Parents Non-central | 866 | 2,173 | 1.630 (1.499, 1.760) |
| SBP | Group 2 | Father Only Central Obesity | 103 | 266 | 1.224 (0.830, 1.617) |
| SBP | Group 3 | Mother Only Central Obesity | 339 | 835 | 1.768 (1.562, 1.974) |
| SBP | Group 4 | Both Parents Central Obesity | 81 | 195 | 1.643 (1.227, 2.060) |
| DBP | Group 1 | Both Parents Non-central | 866 | 2,173 | 1.076 (0.973, 1.179) |
| DBP | Group 2 | Father Only Central Obesity | 103 | 266 | 0.897 (0.587, 1.206) |
| DBP | Group 3 | Mother Only Central Obesity | 339 | 835 | 1.056 (0.894, 1.218) |
| DBP | Group 4 | Both Parents Central Obesity | 81 | 195 | 0.959 (0.632, 1.287) |

*Note.* β represents the model-estimated annual change in the original unit of each outcome. Participant and observation counts are based on records actually included in the corresponding mixed-effects model. BMI = body mass index; WC = waist circumference; SBP = systolic blood pressure; DBP = diastolic blood pressure; CI = confidence interval; CO = central obesity. Group definitions and abbreviations are provided in Supplementary Table S4.

**Supplementary Table S6. Pairwise between-group differences in annual changes in cardiometabolic outcomes by parental central-obesity group**

| **Outcome** | **Contrast** | **Difference in annual change, Δβ (95% CI)** | **Adjusted P value** |
| --- | --- | --- | --- |
| ***Male*** | | | |
| BMI | Group 2 - Group 1 | 0.079 (-0.010, 0.168) | 0.115 |
| BMI | Group 3 - Group 1 | 0.011 (-0.048, 0.070) | 0.998 |
| BMI | Group 4 - Group 1 | 0.009 (-0.119, 0.138) | 1.000 |
| BMI | Group 3 - Group 2 | -0.068 (-0.167, 0.031) | 0.356 |
| BMI | Group 4 - Group 2 | -0.069 (-0.220, 0.082) | 0.789 |
| BMI | Group 4 - Group 3 | -0.001 (-0.137, 0.134) | 1.000 |
| WC | Group 2 - Group 1 | 0.257 (-0.029, 0.542) | 0.104 |
| WC | Group 3 - Group 1 | 0.086 (-0.104, 0.276) | 0.798 |
| WC | Group 4 - Group 1 | 0.255 (-0.151, 0.660) | 0.463 |
| WC | Group 3 - Group 2 | -0.171 (-0.488, 0.146) | 0.639 |
| WC | Group 4 - Group 2 | -0.002 (-0.480, 0.476) | 1.000 |
| WC | Group 4 - Group 3 | 0.169 (-0.259, 0.597) | 0.882 |
| SBP | Group 2 - Group 1 | 0.130 (-0.358, 0.618) | 0.981 |
| SBP | Group 3 - Group 1 | 0.099 (-0.223, 0.420) | 0.962 |
| SBP | Group 4 - Group 1 | -0.189 (-0.867, 0.490) | 0.976 |
| SBP | Group 3 - Group 2 | -0.031 (-0.570, 0.508) | 1.000 |
| SBP | Group 4 - Group 2 | -0.319 (-1.123, 0.486) | 0.880 |
| SBP | Group 4 - Group 3 | -0.287 (-1.003, 0.429) | 0.874 |
| DBP | Group 2 - Group 1 | -0.133 (-0.501, 0.235) | 0.919 |
| DBP | Group 3 - Group 1 | -0.030 (-0.272, 0.213) | 1.000 |
| DBP | Group 4 - Group 1 | 0.148 (-0.362, 0.659) | 0.971 |
| DBP | Group 3 - Group 2 | 0.103 (-0.303, 0.509) | 0.985 |
| DBP | Group 4 - Group 2 | 0.281 (-0.325, 0.887) | 0.779 |
| DBP | Group 4 - Group 3 | 0.178 (-0.361, 0.717) | 0.946 |
| ***Female*** | | | |
| BMI | Group 2 - Group 1 | -0.037 (-0.142, 0.068) | 0.930 |
| BMI | Group 3 - Group 1 | 0.021 (-0.043, 0.084) | 0.948 |
| BMI | Group 4 - Group 1 | -0.031 (-0.146, 0.084) | 0.980 |
| BMI | Group 3 - Group 2 | 0.057 (-0.056, 0.170) | 0.701 |
| BMI | Group 4 - Group 2 | 0.006 (-0.142, 0.154) | 1.000 |
| BMI | Group 4 - Group 3 | -0.052 (-0.174, 0.071) | 0.844 |
| WC | Group 2 - Group 1 | 0.044 (-0.263, 0.352) | 0.999 |
| WC | Group 3 - Group 1 | 0.053 (-0.131, 0.237) | 0.972 |
| WC | Group 4 - Group 1 | 0.143 (-0.189, 0.475) | 0.832 |
| WC | Group 3 - Group 2 | 0.009 (-0.322, 0.339) | 1.000 |
| WC | Group 4 - Group 2 | 0.099 (-0.332, 0.529) | 0.991 |
| WC | Group 4 - Group 3 | 0.090 (-0.263, 0.443) | 0.985 |
| SBP | Group 2 - Group 1 | -0.406 (-0.963, 0.150) | 0.287 |
| SBP | Group 3 - Group 1 | 0.138 (-0.189, 0.466) | 0.845 |
| SBP | Group 4 - Group 1 | 0.014 (-0.572, 0.600) | 1.000 |
| SBP | Group 3 - Group 2 | 0.544 (-0.052, 1.140) | 0.094 |
| SBP | Group 4 - Group 2 | 0.420 (-0.349, 1.189) | 0.626 |
| SBP | Group 4 - Group 3 | -0.124 (-0.748, 0.499) | 0.996 |
| DBP | Group 2 - Group 1 | -0.179 (-0.617, 0.259) | 0.863 |
| DBP | Group 3 - Group 1 | -0.020 (-0.277, 0.238) | 1.000 |
| DBP | Group 4 - Group 1 | -0.117 (-0.578, 0.344) | 0.985 |
| DBP | Group 3 - Group 2 | 0.159 (-0.310, 0.629) | 0.938 |
| DBP | Group 4 - Group 2 | 0.062 (-0.543, 0.667) | 1.000 |
| DBP | Group 4 - Group 3 | -0.097 (-0.588, 0.394) | 0.996 |

*Note.* Δβ is the annual-change estimate for the first group minus that for the second group; positive values indicate a steeper annual increase in the first group. All six pairwise contrasts are shown for each sex and outcome. Confidence intervals and P values were adjusted for multiple pairwise comparisons using the Tukey method. Group definitions and abbreviations are provided in Supplementary Table S4.

**Supplementary Table S7.** Participants at risk, incident events, person-years, and incidence rates according to parental weight-status group and outcome.

| **Outcome** | **Parental weight-status group** | **Participants at risk, n** | **Events, n** | **Person-years** | **Incidence rate per 1,000 person-years** |
| --- | --- | --- | --- | --- | --- |
| Overweight/obesity | Group 1: Both parents NW | 2,044 | 94 | 10,586 | 8.88 |
|  | Group 2: Father only OW/OB | 341 | 32 | 1,662 | 19.25 |
|  | Group 3: Mother only OW/OB | 372 | 31 | 1,883 | 16.46 |
|  | Group 4: Both parents OW/OB | 110 | 16 | 495 | 32.32 |
| Central obesity | Group 1: Both parents NW | 2,108 | 176 | 11,037 | 15.95 |
|  | Group 2: Father only OW/OB | 365 | 60 | 1,790 | 33.52 |
|  | Group 3: Mother only OW/OB | 376 | 47 | 1,921 | 24.47 |
|  | Group 4: Both parents OW/OB | 120 | 26 | 548 | 47.45 |
| Hypertension | Group 1: Both parents NW | 2,016 | 333 | 10,274 | 32.41 |
|  | Group 2: Father only OW/OB | 364 | 66 | 1,794 | 36.79 |
|  | Group 3: Mother only OW/OB | 391 | 65 | 1,977 | 32.88 |
|  | Group 4: Both parents OW/OB | 113 | 26 | 516 | 50.39 |

Notes: Participants at risk, event counts, and person-years were calculated from the complete-case analytical sample used in the corresponding Cox proportional hazards model. Person-years were calculated as the sum of follow-up time from baseline to the first detected incident outcome or the last available follow-up wave, whichever occurred first. Because outcomes were assessed at discrete survey waves, event time was assigned to the first wave at which the outcome was identified. Group 1, both parents with normal weight; Group 2, father only with overweight/obesity; Group 3, mother only with overweight/obesity; Group 4, both parents with overweight/obesity. NW, normal weight; OW/OB, overweight/obesity.

**Table S8. Hazard ratios for incident obesity and hypertension associated with parental central obesity.**

| **Parental Obesity Group** | **Obesity Incidence** | **Central Obesity Incidence** | **Hypertension Incidence** |
| --- | --- | --- | --- |
| **Overall** |  |  |  |
| ****G1: Neither Central Obesity**** | ****1.00 (Reference)**** | ****1.00 (Reference)**** | ****1.00 (Reference)**** |
| ****G2: Only Father Central Obesity**** | 2.24 (1.35, 3.72) | 1.23 (0.84, 1.80) | 1.04 (0.74, 1.47) |
| ****G3: Only Mother Central Obesity**** | 1.77 (1.21, 2.58) | 1.23 (0.92, 1.63) | 1.04 (0.83, 1.29) |
| ****G4: Both Parents Central Obesity**** | 6.10 (3.84, 9.70) | 1.76 (1.18, 2.61) | 1.64 (1.14, 2.34) |
| **Boys** |  |  |  |
| ****G1: Neither Central Obesity**** | ****1.00 (Reference)**** | ****1.00 (Reference)**** | ****1.00 (Reference)**** |
| ****G2: Only Father Central Obesity**** | 1.67 (0.82, 3.40) | 0.96 (0.54, 1.70) | 1.10 (0.70, 1.70) |
| ****G3: Only Mother Central Obesity**** | 2.17 (1.36, 3.47) | 0.96 (0.62, 1.49) | 1.18 (0.89, 1.57) |
| ****G4: Both Parents Central Obesity**** | 5.22 (2.76, 9.86) | 2.01 (1.08, 3.75) | 1.58 (0.93, 2.68) |
| ****Girls**** |  |  |  |
| ****G1: Neither Central Obesity**** | ****1.00 (Reference)**** | ****1.00 (Reference)**** | ****1.00 (Reference)**** |
| ****G2: Only Father Central Obesity**** | 4.20 (1.89, 9.32) | 1.70 (0.98, 2.93) | 0.85 (0.48, 1.52) |
| ****G3: Only Mother Central Obesity**** | 1.30 (0.67, 2.54) | 1.36 (0.92, 2.01) | 0.80 (0.56, 1.14) |
| ****G4: Both Parents Central Obesity**** | 8.23 (3.98, 17.03) | 1.87 (1.11, 3.14) | 1.59 (0.96, 2.64) |
| *****P*_interaction (sex*_**_Parental Obesity_**_)_**** | **0.214** | 0.677 | 0.317 |

Covariates included sex(for overall model), offspring baseline age, ethnicity, region, parental age, smoking status, drinking status, and education at the survey year.

**Supplementary Table S9.** Participants at risk, incident events, person-years, and incidence rates according to parental central-obesity group and outcome.

| **Outcome** | **Parental central-obesity group** | **Participants at risk, n** | **Events, n** | **Person-years** | **Incidence rate per 1,000 person-years** |
| --- | --- | --- | --- | --- | --- |
| Overweight/obesity | Group 1: Both parents non-central obesity | 1,847 | 83 | 9,628 | 8.62 |
|  | Group 2: Father only central obesity | 209 | 20 | 1,071 | 18.67 |
|  | Group 3: Mother only central obesity | 654 | 44 | 3,268 | 13.46 |
|  | Group 4: Both parents central obesity | 157 | 26 | 659 | 39.45 |
| Central obesity | Group 1: Both parents non-central obesity | 1,909 | 164 | 10,027 | 16.36 |
|  | Group 2: Father only central obesity | 224 | 36 | 1,153 | 31.22 |
|  | Group 3: Mother only central obesity | 675 | 74 | 3,420 | 21.64 |
|  | Group 4: Both parents central obesity | 161 | 35 | 696 | 50.29 |
| Hypertension | Group 1: Both parents non-central obesity | 1,828 | 300 | 9,378 | 31.99 |
|  | Group 2: Father only central obesity | 221 | 38 | 1,137 | 33.42 |
|  | Group 3: Mother only central obesity | 675 | 115 | 3,343 | 34.40 |
|  | Group 4: Both parents central obesity | 160 | 37 | 703 | 52.63 |

Notes: Participants at risk, event counts, and person-years were calculated from the complete-case analytical sample used in the corresponding Cox proportional hazards model. Person-years were calculated as the sum of follow-up time from baseline to the first detected incident outcome or the last available follow-up wave, whichever occurred first. Because outcomes were assessed at discrete survey waves, event time was assigned to the first wave at which the outcome was identified. Group 1, both parents without central obesity; Group 2, father only with central obesity; Group 3, mother only with central obesity; Group 4, both parents with central obesity.
